# Associations Between Resting Heart Rate, Resting Blood Pressure, Psychological Variables and Pain Processing in Chronic Whiplash-Associated Disorder: A Cross-Sectional Study

**DOI:** 10.1093/pm/pnac075

**Published:** 2022-05-19

**Authors:** Liam White, Ashley D Smith, Scott F Farrell

**Affiliations:** School of Health and Rehabilitation Sciences, The University of Queensland, Brisbane, Australia; Department of Clinical Neurosciences, Cumming School of Medicine, University of Calgary, Calgary, Canada; RECOVER Injury Research Centre, NHMRC Centre for Research Excellence: Better Health Outcomes for Compensable Injury, The University of Queensland, Brisbane, Australia; Menzies Health Institute Queensland, Griffith University, Gold Coast, Australia

**Keywords:** Whiplash Injuries, Heart Rate, Blood Pressure, Pain Threshold, Chronic Pain, Neck Pain

## Abstract

**Objective:**

Autonomic nervous system dysfunction has been implicated in chronic whiplash-associated disorder (WAD). However, the relationship between autonomic variables (e.g., resting heart rate and blood pressure) and clinical factors in chronic WAD is not well understood. This study sought to examine the associations between resting heart rate, resting blood pressure, pain processing and psychological variables in chronic WAD and in pain-free controls.

**Design:**

Secondary analysis of a cross-sectional study.

**Setting:**

University clinical research laboratory.

**Subjects:**

Thirty-six people with chronic WAD Grade II (mean [SD] age 40.1 [14.6] years, 28 females) and 25 pain-free controls (35.6 [13.0] years, 17 females).

**Methods:**

Participants had resting heart rate, systolic and diastolic blood pressure measured. Pain processing measures comprised: (i) pain pressure threshold at the cervical spine, hand and leg, (ii) temporal summation at the cervical spine and hand, and (iii) conditioned pain modulation. Psychological outcomes included measures of kinesiophobia, pain catastrophizing and post-traumatic stress symptoms. Correlations between autonomic variables, pain processing and psychological variables were determined (*P* < .05, 5% FDR).

**Results:**

No significant correlations between autonomic and pain processing variables, or autonomic and psychological variables were found in the chronic WAD group. In the control group, diastolic blood pressure was positively correlated with cervical spine pressure pain threshold (r = 0.53, *P* = .007).

**Conclusions:**

An association between blood pressure and pain sensitivity was observed in the control group but not the chronic WAD group. Such an association appears to be disrupted in chronic WAD, which may infer involvement of autonomic pathways in the pathophysiology of this condition.

## Introduction

Approximately 50% of people that sustain a whiplash injury will develop ongoing pain and disability, termed chronic whiplash-associated disorder (WAD) [1]. Current guideline-based treatment for WAD comprises reassurance, advice to remain active and exercises [2]. However, this approach typically demonstrates small to moderate effect sizes [3, 4], which may be a result of an incomplete understanding of the pathophysiological mechanisms underlying WAD [5, 6].

Chronic WAD appears to be underpinned by both biological and psychological pathways. Somatosensory changes observed in chronic WAD patients include dysfunction of pain processing, such as reduced pain pressure thresholds (PPT) [7–9], increased sensitivity to repeated noxious stimuli (temporal summation [TS] of pain) [7, 10] and impaired diffuse noxious inhibitory control (conditioned pain modulation [CPM]) [11]. In addition to altered pain processing, chronic WAD patients often present with psychological issues [12] including pain catastrophizing and kinesiophobia, as well as post-traumatic stress symptoms [1]. There is emerging evidence of altered autonomic function in people with chronic WAD, in the form of lower resting heart rate variability [13]. Differences in autonomic function in chronic WAD are potentially clinically important, as they may reflect mechanistic processes that contribute to patient symptoms, such as overlapping pathways common to cardiovascular control and pain sensory processing [14, 15].

In pain-free populations, relationships between autonomic variables and measures of pain processing have been identified. Higher resting blood pressure (BP) has been observed to be associated with reduced sensitivity to noxious stimuli (i.e., higher pressure [16], thermal [17] and ischemic [17] pain thresholds) and lower TS [18]. This reciprocal relationship between BP and pain sensitivity is thought to reflect baroreceptor reflex function, whereby higher BP increases baroreceptor stimulation, which subsequently activates descending pain inhibitory pathways, as well as endogenous opioid and noradrenergic mechanisms [19, 20]. However, this “hypertension-associated hypoalgesia” found in pain-free populations [20] has been observed to be absent in chronic temporomandibular pain [17] and reversed in chronic back pain [16, 18] patients.

There also appears to be relationships between autonomic variables and psychological factors in pain-free populations. For instance, post-traumatic stress symptoms [21] are positively correlated with resting BP and psychological distress is associated with altered heart rate variability [22], while resting heart rate (HR) increases in acute stress [23] and appears to reduce in chronic stress [24, 25]. These relationships warrant examination in chronic WAD, given the association between psychological factors and poor outcome after whiplash injury (e.g., post-traumatic stress symptoms [1, 26]).

Associations between autonomic variables and other clinical factors in chronic WAD, such as pain processing and psychological symptoms, have not been fully explored. One study found that resting HR and heart rate variability were unrelated to CPM and PPT (trapezius and quadriceps muscles) in people with chronic WAD and pain-free controls [27], whereas another study reported resting HR and trapezius muscle PPT to be positively correlated in chronic WAD patients but not in pain-free individuals [28]. However, other studies examining chronic back pain found higher resting systolic and diastolic blood pressure (SBP, DBP) to be related to lower PPT [16] and facilitated TS [18] in people with chronic back pain, contrary to the nature of these relationships in pain-free populations. Associations between autonomic variables and psychological factors in chronic WAD have not been comprehensively investigated. Koenig et al. [13] found lower heart rate variability was associated with higher pain catastrophizing in chronic WAD, while another study found no association between resting HR, kinesiophobia, and pain catastrophizing in chronic neck pain [29].

In pain-free populations, there are relationships between autonomic variables, pain processing and psychological variables, including “hypertension-associated hypoalgesia” [16]. However, there is evidence of these relationships being disrupted in chronic temporomandibular [17] and back pain [16, 18], which may reflect altered neurophysiological pathways underpinning symptoms. In chronic WAD these relationships are not well understood. Improving knowledge of pathophysiological mechanisms underlying chronic pain conditions such as WAD may allow development of treatment strategies targeting such pathways. More broadly, the relationship between autonomic cardiovascular variables and chronic pain conditions is of particular interest, given the observed relationships between cardiovascular disease and chronic pain conditions [30, 31]. This study sought to characterize associations between resting HR and BP and clinical outcomes including CPM, TS, PPT, post-traumatic stress symptoms, pain catastrophizing, and kinesiophobia in people with chronic WAD and in pain-free controls.

## Methods

### Ethics Statement

Ethical clearance for this study was granted by Griffith University human research ethics committee. All participants provided written informed consent. The study was conducted in accordance with ethical standards laid down in the Declaration of the Helsinki. This study is a secondary analysis using baseline data from a previous study that examined exercise-induced hypoalgesia in chronic WAD [32].

### Participants

Participants (aged 18–65 years) were recruited with chronic WAD Grade II (musculoskeletal neck pain, no fracture/dislocation, or neurological deficit [33]) and duration of pain between 3 months and 10 years. Participants had ≥ 4/10 neck pain intensity on a numerical rating scale (NRS) or indicated at least moderate limitation of daily activities due to pain (as per Item 8 of the SF-36 questionnaire). These patients were compared with age- and sex-matched controls.

People with WAD IV (fracture or dislocation), WAD III (with neurological deficits in the upper limbs), history of migraine headaches, history of surgery to the neck, metabolic disease (e.g., diabetes), neurological disease (e.g., multiple sclerosis), cardiovascular diagnoses (including hypertension), or psychiatric (e.g., depression) diseases were excluded from the study. Individuals who were pregnant, breastfeeding, or unable to discontinue analgesic/anti-inflammatory medications for 48 hours prior to participation were excluded. As the primary study [32] included an exercise component, individuals who screened positive on the Physical Activity Readiness Questionnaire [34] indicating a need to consult a medical practitioner prior to participating in physical activity were also excluded.

### Clinical Questionnaires

Basic demographic data were collected. In the chronic WAD group, duration of symptoms was recorded and average pain intensity over the last 24 hours was assessed using a 100 mm Visual Analogue Scale (VAS). All participants completed the Neck Disability Index (NDI) [35], the Tampa Scale of Kinesiophobia (TSK) [36] and the Pain Catastrophizing Scale (PCS) [37], with the chronic WAD group also completing the Post-traumatic Stress Disorder Checklist (PCL-S) [38]. The NDI gives a percentage score representing neck pain-related disability [35], with scores > 28% indicating moderate-severe disability [8]. The TSK assesses fear of movement as a score from 17–68 [36] with scores ≥ 37 considered a significant level of kinesiophobia [39]. The PCL-S assesses post-traumatic stress symptoms as a score of 17–85 [38] with scores above 30 considered to indicate significant symptoms in general populations [40]. The PCS assesses catastrophic thinking about pain as a score from 0–52 with scores ≥ 30 suggesting clinically relevant catastrophizing [37].

### Resting Heart Rate and Blood Pressure

Measures of resting HR, SBP and DBP were taken with the participant seated after a 5-minute quiet resting period [41]. HR was measured with a POLAR HR monitor chest strap (RS300X, Polar, Finland) and BP was taken manually using a mercury sphygmomanometer (Standby model, W.A. Baumanometer Co Inc., USA) and then verified with an automatic BP monitor (IA1B model, Omron, Japan). For descriptive purposes, resting HR, SBP, and DBP were considered with reference to normative ranges of 60–100 beats per minute (BPM) [42], 90–140 mmHg and 60–90 mmHg, respectively [43, 44]. The HR and BP measures were performed prior to the pain processing measures.

### Pain Processing

Measures of PPT, TS, and CPM were taken with standardized verbal instructions as per Rolke et al. [45]. PPT was assessed using a Somedic SENSELab AB (Fastra, Sweden) algometer with a 1 cm^2^ probe, with pressure applied at a rate of 40 kPa/s on the cervical spine (right C5-6 articular pillar) with the patient in a prone position. Participants were advised to press a handheld switch at the first moment that the sensation changed from one of pressure to one of pressure and pain. PPTs were also performed at two distal locations: the right dorsum of the hand between 2nd and 3rd metacarpals and the left tibialis anterior, both done with the participant seated. These sites were selected to allow assessment of localized and widespread hyperalgesia. PPT has previously been assessed at these locations in studies in WAD [8, 46]. Triplicate measures were taken for each site (20 s stimulus interval), with the average recorded as PPT for that location.

To assess TS, wind-up ratio was calculated using the protocol described by Rolke et al. [45]. Briefly, perceived pain intensity (0–100 numerical rating scale [NRS]) of a single 256 mN pinprick stimulus (MRC Systems GmbH, Heidelberg, Germany) was compared with perceived pain intensity (0–100 NRS) of a series of ten of the same pinprick stimulus of the same physical intensity (1/s applied within an area of 1 cm^2^). This was repeated three times, and the average NRS after 10 pin pricks was divided by the average NRS after one pin prick to determine the wind-up ratio. To assess TS at local and distal sites, this procedure was performed over the C5-C6 neck region in prone and on the dorsum of the hand between 2nd and 3rd metacarpals in sitting.

For assessment of CPM, the conditioning stimulus was immersion of the right hand in thermostatically controlled 5°C water for 2 minutes. The test stimulus was PPT at left tibialis anterior performed as described above. Both of these stimuli have been used in CPM in prior research [47]. Baseline PPT was calculated as the average of triplicate measures as described above, and PPT during the conditioning stimulus was calculated as the average of four measures taken in the presence of the conditioning stimulus (immediately, 30 seconds, 60 seconds, and 90 seconds following immersion of the right hand in ice water). The CPM value was determined by subtracting the average of the PPT measurements taken during application of the conditioning stimulus from the average of the baseline PPT values, quantified as kPa and as a percentage of baseline PPT. This is consistent with recommendations by Yarnitsky et al. [48], such that a negative CPM value denotes inhibition of test stimulus pain in the presence of the conditioning stimulus. During the application of the conditioning stimulus, time until first onset of pain, duration of immersion lasted, and peak pain intensity rating (NRS) were recorded.

### Statistical Analysis

Data were analyzed using IBM SPSS (v26, Armonk, NY, USA). Normality was determined for each variable by visual inspection and Shaprio-Wilk testing. For descriptive purposes, outcomes were summarizd as mean and standard deviation or median and interquartile range as appropriate to distribution. Demographic, clinical, questionnaire and pain processing data were compared between groups using independent *t*-tests or Mann-Whitney *U* tests as appropriate to distribution (*P* < .05). Sex was compared between groups using a 2×2 table (χ^2^). Correlations between the primary outcome measures (resting HR, SBP, and DBP) and the psychological and pain processing variables were determined with Pearson or Spearman correlations (as appropriate to distribution), with Benjamini-Hochberg corrections to account for multiple comparisons at 5% false discovery rate (FDR). In order to detect a moderate correlation of r = 0.45 at 80% power, 36 chronic WAD participants were required [49].

## Results

### Demographic and Clinical Characteristics

Thirty-six participants with chronic WAD and 25 controls were included. Demographic and outcome measure descriptive data are presented in Table 1. Duration of symptoms ranged from 3 months to 10 years (median [IQR] 22 [36] months) in the chronic WAD group. Participants in the chronic WAD group reported average pain intensity ranging from 0 to 73 mm (median [IQR] 51 [32]). The chronic WAD group had significantly higher scores than controls on the NDI, TSK, and PCS. In the chronic WAD group, 26 (72.2%) patients had scores indicating moderate-severe neck pain-related disability (> 28%) on the NDI (mean [SD] 36.4 [13.4]), while 20 (55.6%) patients had scores on the TSK (≥ 37) indicating high levels of kinesiophobia (mean [SD] 37 [8.3]). Twelve participants (33.3%) in the chronic WAD group had PCL-S scores consistent with significant post-traumatic stress symptoms (≥30) (median [IQR] 23 [17]). One participant (2.8%) in the chronic WAD group did not complete the PCL-S questionnaire. Five participants (13.9%) in the chronic WAD group had PCS scores consistent with significant pain catastrophizing (≥30) (median [IQR] 11 [14]).

**Table 1.** Demographic, clinical and resting heart rate and blood pressure data for chronic whiplash-associated disorder (WAD) and control groups

Characteristic	Chronic WAD	Controls	Test Statistic	*P*
	N = 36	N = 25		
Age (years), Mean (SD)	40.1 (14.6)	35.6 (13.0)	t = 1.25	.22
Sex (female), n (%)	28 (78)	17 (68)	χ^2^ = 0.73	.39
Body Mass Index (kg/m^2^), Mean (SD)	24.6 (5.0)	23.9 (3.8)	t = 0.57	.57
Duration (months), Median (IQR)	22 (36)	…	…	…
Visual Analogue Scale (0–100 mm), Median (IQR)	51 (32)	…	…	…
Neck Disability Index (%), Median (IQR)	37 (20)	2 (6)	z = 6.62	**<.001**
Pain Catastrophizing Scale, Median (IQR)	11 (14)	5 (12)	z = 3.30	**.001**
Post-traumatic Stress Disorder Checklist, Median (IQR)	23 (17)	…	…	…
Tampa Scale of Kinesiophobia, Mean (SD)	37 (8)	27 (6)	t = 5.13	**<.001**
Resting Heart Rate (beats/minute), Mean (SD)	77.7 (13.5)	75.7 (11.2)	t = 0.59	.56
Resting Systolic Blood Pressure (mmHg), Mean (SD)	115.4 (11.5)	116.1 (9.9)	t = −0.27	.79
Resting Diastolic Blood Pressure (mmHg), Mean (SD)	76.8 (10.1)	75.2 (8.3)	t = 0.66	.51

Bolded *P* values are significant.

### Autonomic Variables

Resting HR, SBP, and DBP for the chronic WAD and control groups can be seen in Table 1. There were no group differences in any of these variables. Thirty participants in the chronic WAD group (83.3%) and 24 participants in the control group (96.0%) had resting HR values between 60 and 100 BPM. Four chronic WAD patients (11.1%) and one control (4.0%) had HR below this range, while two chronic WAD patients (5.6%) had HR above this range. Resting SBP was between 90 and 140 mmHg for 35 participants in the chronic WAD group (97.2%) and for 20 participants in the control group (80.0%). One chronic WAD participant (2.8%) had SBP below this range and five controls (20.0%) had SBP above this range. Resting DBP was between 60 and 90 mmHg for 32 participants in the chronic WAD group (88.9%) and for 24 participants in the control group (96.0%). One chronic WAD patient (2.8%) had resting DBP below this range, while three chronic WAD patients (8.3%) and one control (4.0%) were above the range.

### Pain Processing

Summary statistics for pain processing outcomes (PPT, TS, and CPM) can be found on Table 2. At tibialis anterior, PPT was lower in the chronic WAD group than the control group (Chronic WAD: median [IQR] 352.2 [191.7] kPa; Controls: 467.0 [218.5] kPa; z = −3.05, *P* = .002). There were no other significant group differences for pain processing variables. Three participants in the chronic WAD group (8.3%), and three participants in the control group (12.0%) reported no pain during the single pin prick (score of 0 NRS) for wind-up ratio at the hand, so a ratio could not be calculated with zero as a denominator [45]. This was also applicable for two participants in the chronic WAD group (5.6%) and two participants in the control group (8.0%) for the wind-up ratio of the C5-C6 area of the cervical spine.

**Table 2.** Pain processing data for chronic whiplash-associated disorder (WAD) and pain groups

Measure	Chronic WAD	Controls	Test Statistic	*P*
	N = 36	N = 25		
Pain Pressure Threshold at Hand (kPa), Mean (SD)	260.5 (108.8)	307.7 (112.8)	t = −1.64	.11
Pain Pressure Threshold at Cervical Spine (kPa), Median (IQR)	240.8 (143.3)	268.0 (176.2)	z = −1.22	.22
Pain Pressure Threshold at Tibialis Anterior (kPa), Median (IQR)	352.2 (191.7)	467.0 (218.5)	z = −3.05	**.002**
Wind Up Ratio Hand, Median (IQR)	2.6 (3.1)	2.0 (2.1)	z = 1.46	.14
Wind Up Ratio at Cervical Spine, Median (IQR)	2.0 (1.7)	1.8 (1.1)	z = 0.67	.51
Conditioned Pain Modulation (kPa), Median (IQR)	−118.3 (173.3)	−119.8 (245.5)	z = −0.29	.77
Conditioned Pain Modulation (% of baseline), Median (IQR)	30.0 (70.7)	31.9 (46.2)	z = −0.87	.39

Bolded *P* values are significant.

During the CPM assessment, the time until pain onset during the conditioning stimulus (hand in ice water) did not differ between groups (Chronic WAD: median [IQR] 9 [6] s; Controls: 10 [7] s; z = -0.71, *P* = .48). Time lasted with conditioning stimulus did not differ between groups (Chronic WAD: median [IQR] 120 [0] s; Controls: median [IQR] 120 [0] s; z = −1.37, *P* = 0.17). Eight participants in the chronic WAD group (22.2%) and two in the control group (8.0%) did not tolerate the full 120 seconds of conditioning stimulus time. The NRS values for peak pain intensity given during conditioning stimuli application ranged from 4 to 10/10 in the chronic WAD group and 3–10/10 in the control group (Chronic WAD: median [IQR] 9 [2]; Controls: 8 [2]; z = 1.28, *P* = .20).

### Correlations Between Clinical Characteristics, Pain Processing, and Autonomic Variables

**Figure 1.**
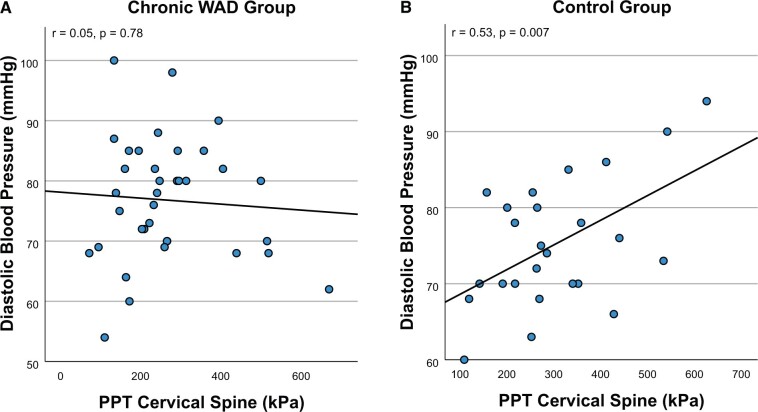
Scatterplots illustrating relationships between resting diastolic blood pressure and pressure pain threshold (PPT) at the cervical spine in (**A**) people with chronic whiplash-associated disorder (WAD) and (**B**) pain-free controls. In the control group (r = 0.53, *P* = .007), this correlation was significant following correction for multiple comparisons (5% false discovery rate).

There were no significant correlations between resting HR, SBP or DBP and pain catastrophizing or kinesiophobia in either group, while in the chronic WAD group there were no significant correlations between PCL-S, NDI, VAS or duration of symptoms and resting HR, SBP or DBP (Table 3, Supplementary Data Figures S1 and S2). Associations between resting HR, SBP or DBP and pain processing variables can be seen in Table 4 and Supplementary Data Figures S3 and S4. There were no significant correlations following correction for multiple comparisons (5% FDR) in the chronic WAD group. One positive correlation between resting DBP and PPT at the cervical spine in the control group (r = 0.53, *P* = .007) survived correction for multiple comparisons (Figure 1).

**Table 3.** Correlations between psychological and clinical characteristics and autonomic variables

	PCS	PCL-S	TSK	VAS	NDI	Duration
*Chronic WAD* (*N = *36)						
Resting HR	−0.16	−0.03	0.04*	−0.25	0.03*	0.06
	(0.34)	(0.87)	(0.84)	(0.14)	(0.87)	(0.74)
Resting SBP	0.14	−0.13	−0.18*	−0.25	−0.19*	−0.03
	(0.43)	(0.46)	(0.29)	(0.14)	(0.27)	(0.88)
Resting DBP	0.00	−0.14	−0.07*	−0.30	−0.11*	−0.12
	(0.99)	(0.42)	(0.67)	(0.08)	(0.51)	(0.47)
*Controls* (*N = *25)						
Resting HR	−0.05	…	0.40*	…	…	…
	(0.83)		(0.05)			
Resting SBP	−0.07	…	0.29*	…	…	…
	(0.74)		(0.15)			
Resting DBP	0.13	…	0.22*	…	…	…
	(0.54)		(0.29)			

Data are presented as correlation coefficient (*P* values). Spearman correlations used unless indicated otherwise. No correlations were significant following correction for multiple comparisons (FDR 5%). HR = heart rate; SBP = systolic blood pressure; DBP = diastolic blood pressure; VAS = Visual Analogue Scale; NDI = Neck Disability Index; PCS = Pain Catastrophizing Scale; TSK = Tampa Scale of Kinesiophobia; PCL-S = Posttraumatic Stress Disorder Checklist.

* Pearson correlation rather Spearman correlation used, as appropriate to distribution.

**Table 4.** Correlations between pain processing outcomes and autonomic variables

	PPT C-Spine	PPT Hand	PPT Tib Ant	WUR C-Spine	WUR Hand	CPM (kPa)	CPM (%)
*Chronic WAD* (*N = *36)							
Resting HR	−0.07	−0.07*	−0.09	−0.01	0.41	0.00	0.02
	(0.68)	(0.70)	(0.62)	(0.98)	(0.02)	(0.99)	(0.92)
Resting SBP	0.12	0.27*	0.19	0.08	−0.05	−0.10	0.03
	(0.51)	(0.11)	(0.26)	(0.66)	(0.77)	(0.57)	(0.85)
Resting DBP	0.05	0.16*	0.11	0.14	0.05	0.01	0.12
	(0.78)	(0.34)	(0.51)	(0.42)	(0.77)	(0.97)	(0.42)
*Controls* (*N = *25)							
Resting HR	−0.10*	−0.21*	−0.04	0.14	−0.04	−0.13*	−0.12*
	(0.64)	(0.31)	(0.86)	(0.52)	(0.88)	(0.53)	(0.57)
Resting SBP	0.27	0.44*	0.32	0.08	−0.15	0.23*	0.21*
	(0.20)*	(0.03)	(0.12)	(0.71)	(0.52)	(0.26)	(0.31)
Resting DBP	**0.53** *	0.46*	0.18	−0.09	−0.17	0.08*	0.05*
	**(0.007)**	(0.02)	(0.38)	(0.69)	(0.46)	(0.71)	(0.80)

Data are presented as correlation coefficient (*P* values). Bold indicates significant following correction for multiple comparisons (FDR 5%). HR = heart rate; SBP = systolic blood pressure; DBP = diastolic blood pressure; PPT = pressure pain threshold; WUR = wind-up ratio; CPM = conditioned pain modulation (quantified in kPa and as % of baseline PPT).

* Pearson correlation rather Spearman correlation used, as appropriate to distribution.

## Discussion

Following corrections for multiple comparisons, the present study did not find significant correlations between resting HR or BP and pain processing variables (CPM, TS, PPT) or psychological variables (post-traumatic stress symptoms, pain catastrophizing, kinesiophobia) in people with chronic WAD. However, in the control group a positive correlation was noted between resting DBP and PPT at the cervical spine. Taken together, these findings suggest that higher resting BP may be associated with lower sensitivity to blunt pressure noxious stimuli in pain-free individuals; however in chronic WAD, this association appears to be absent.

Altered pain processing has clinical significance in WAD. Thermal and mechanical hyperalgesia is present in acute and chronic WAD [1, 8], while in patients with chronic WAD, impairments in CPM [11] and TS [10] have been observed. In the present study, we found some evidence of widespread hyperalgesia in chronic WAD (lower PPTs at tibialis anterior compared with controls); however, group differences in PPT at the neck and hand did not reach significance (*P* = .11 and .22, respectively). We suggest that in a larger sample, these differences may have reached significance, in line with a prior meta-analysis revealing lower PPTs at neck and upper limb sites in chronic WAD [50].

In our data, higher resting DBP was associated with higher cervical spine PPTs in pain-free controls. This is consistent with other studies that reported resting BP to be associated with lower pain sensitivity, in the absence of chronic pain conditions. For instance, in pain-free subjects, higher resting BP was associated with greater upper limb ischemic pain threshold, thermal and ischemic pain tolerance [17], as well as reduced sensitivity to a finger pressure pain test and an ischemic forearm measure [16]. Furthermore, higher resting BP was associated with lower TS to a thermal stimulus [18]. In animal studies, inducing hypertension by drug, diet, or renal artery clipping results in hypoalgesia and lowering BP in genetically hypertensive rats reverses their usual hypoalgesia [51]. This reciprocal relationship between BP and pain sensitivity is thought to be mediated by the baroreceptor reflex, whereby greater BP increases baroreceptor stimulation which subsequently activates descending pain inhibitory pathways [19].

In people with chronic pain conditions, there is evidence that: (i) this “hypertension-associated hypoalgesia” relationship (normally present in pain-free populations [20]) is reversed [16, 18], or (ii) the relationship between these variables is absent [17]. In line with (i), Bruehl et al. [16] found higher resting SBP to be associated with higher pain thresholds in pain-free individuals and lower pain thresholds in low back pain patients. Similarly, Chung et al. [18] reported higher BP to be associated with lower TS of thermal pain in pain-free controls, and the reverse relationship in patients with chronic back pain. In line with (ii), Maixner et al. [17] found that higher BP was associated with lower thermal and ischemic pain sensitivity in pain-free controls, however this relationship was absent in temporomandibular disorder. Similarly, we observed a relationship between DBP and PPT in controls, which was not present in the chronic WAD group. Disruption of the relationship between resting BP and pain sensitivity could be due to impaired descending pain inhibitory pathways that may occur in chronic pain conditions [52] or altered baroreceptor sensitivity as has been found in chronic musculoskeletal pain [51]. Interestingly, we found no group differences in CPM (quantified in kPa or as a % change from baseline) between the chronic WAD and control groups, nor associations between CPM and resting SBP or DBP, suggesting our data is not consistent with the proposition that impaired descending pain inhibition accounts for the disruption of the relationship between resting BP and pain sensitivity. The present study did not investigate baroreceptor reflex function; however, Chung and Bruehl [18] found that spontaneous baroreflex sensitivity was associated with lower TS in pain-free individuals but not in back pain patients, implying that baroreceptor reflex function may explain their observed association between higher BP and reduced TS in controls (and reversal of this relationship in back pain patients). Similarly, in fibromyalgia impaired baroreflex sensitivity was associated with increased pain sensitivity assessed by cold pressor test [53]. Taken together, it appears that the reciprocal relationship between BP and pain sensitivity present in pain-free individuals is absent or disrupted in chronic WAD patients—although it should be noted that in our data, only the correlation between PPT cervical spine and DBP was significant, while the correlations between PPT hand & SBP, and PPT hand and DBP did not survive correction for multiple comparisons (*P* = .03 and .02, respectively). While the physiological pathways underlying dysfunction of baroreceptor reflex-mediated pain inhibition in chronic pain conditions are not well understood [51], progressing knowledge of the relationships between these variables in conditions like chronic WAD will advance understanding of the mechanisms underpinning such conditions.

Our analysis did not identify significant relationships between resting HR and pain processing outcomes in either the chronic WAD or control groups, although the positive correlation between resting HR and TS (wind-up ratio) in the chronic WAD group approached significance but did not survive correction for multiple comparisons (r = 0.41, *P* = .02). De Kooning et al. [28] found a moderate correlation (r = 0.48, *P* = .008) between resting HR and trapezius PPT in chronic WAD, although—consistent with the present study—they reported no associations between HR and CPM and PPT at a lower limb site (quadriceps). The association between resting HR and trapezius PPT observed by De Kooning et al. [28] could be related to altered autonomic function in chronic WAD, as a relationship between chronic pain and higher resting HR has been reported, which may imply increased sympathetic nervous system activation and reduced parasympathetic counteraction [54]. However, this association is not consistently reported, as another study [27] examining chronic WAD and controls found that HR during experimental pain testing was not correlated with PPTs (trapezius and quadriceps) or CPM. No prior study has examined the relationship between TS and resting HR and our study would suggest that there may be no association. Overall, our study findings suggest there is no association between resting HR and pain processing measures, and evidence for an association between these variables is conflicting in the literature.

Psychological variables have previously been noted to be associated with resting HR and BP. For example, pain catastrophizing (in mixed chronic musculoskeletal pain populations) and post-traumatic stress symptoms (in general populations) have been shown to be related to higher resting BP [21]. Resting HR has been observed to be raised in acute stress [23] and reduced in chronic stress [24, 25]. Interestingly, our study found no correlations between resting HR or BP and psychological measures (PCS, TSK, PCL-S) in either controls or chronic WAD patients. This is in agreement with prior investigations in chronic back pain [55] and neck pain [29], reporting no associations between resting HR and psychological factors. We also found no correlations between resting BP and psychological outcome measures. This is contrary to another study which found small correlations between pain catastrophizing and resting SBP and resting DBP in a mixed chronic musculoskeletal pain population [56]. It is important to consider that while the chronic WAD participants reported substantial levels of kinesiophobia, most of the chronic WAD group participants (24/36) did not have PCL-S scores consistent with significant levels of post-traumatic stress or significant pain catastrophizing (30/36)—nor did any of the pain-free controls—which may have impacted our capacity to assess the relationship between these psychological factors and pain processing measures.

This study has several limitations. As a secondary analysis using the baseline data taken for a previous study [32], the sample size of the present study was powered to find a moderate correlation. However, the decision to strictly correct for multiple comparisons (5% FDR) due to the numerous correlations performed means there is (accordingly) an increased risk of type II errors, particularly in the control group (smaller sample size). Additionally, the physiotherapist researcher that performed the laboratory assessments was not blinded to the WAD/control status of the participants. Future research on this topic is required utilizing: (i) larger sample sizes and longitudinal designs, to allow exploration of the temporal development of potential aberration of the relationship between pain sensitivity and autonomic variables (such as BP) after a whiplash injury, (ii) participants with a wider range of scores on the psychological measures, (iii) assessments of baroreflex sensitivity [18, 53], HR and BP reactivity and variability [28, 29], and (iv) longitudinal study designs to characterize relationships between HR, BP, pain processing, and psychological factors in the acute phase following whiplash injury and subsequently through the transition to chronic pain.

In conclusion, we found no significant correlations between resting HR or BP and pain processing variables (CPM, TS, PPT) or psychological variables (post-traumatic stress symptoms, pain catastrophizing, kinesiophobia) in people with chronic WAD. However, in the control group a positive correlation was noted between resting DBP and PPT at the cervical spine. These data suggest that the relationship between higher BP and lower pain thresholds seen in pain-free individuals may not be present in chronic WAD. Furthermore, no associations were noted between resting HR and pain-processing in chronic WAD or controls, nor between psychological variables and resting HR or BP in either group. These data provide novel insight on the potential involvement of autonomic pathways in the pathophysiology of chronic WAD.

## Supplementary Material

pnac075_Supplementary_Data

## Notes

Funding sources: This research received no specific grant from any funding agency in the public, commercial, or not-for-profit sectors.

Ethics statement: All procedures performed in this study were in accordance with the institutional research committee and with the 1964 Helsinki Declaration and its later amendments.
