# Supplementary material for: Associations Between Resting Heart Rate, Resting Blood Pressure, Psychological Variables and Pain Processing in Chronic Whiplash-Associated Disorder: A Cross-Sectional Study
Source: Pain Med. 2022 May 19;23(11):1882–90. doi: 10.1093/pm/pnac075 (PMC9629357; doi:10.1093/pm/pnac075)
Supplement: pnac075_Supplementary_Data [file pnac075_supplementary_data.pdf]

Supplementary Figures

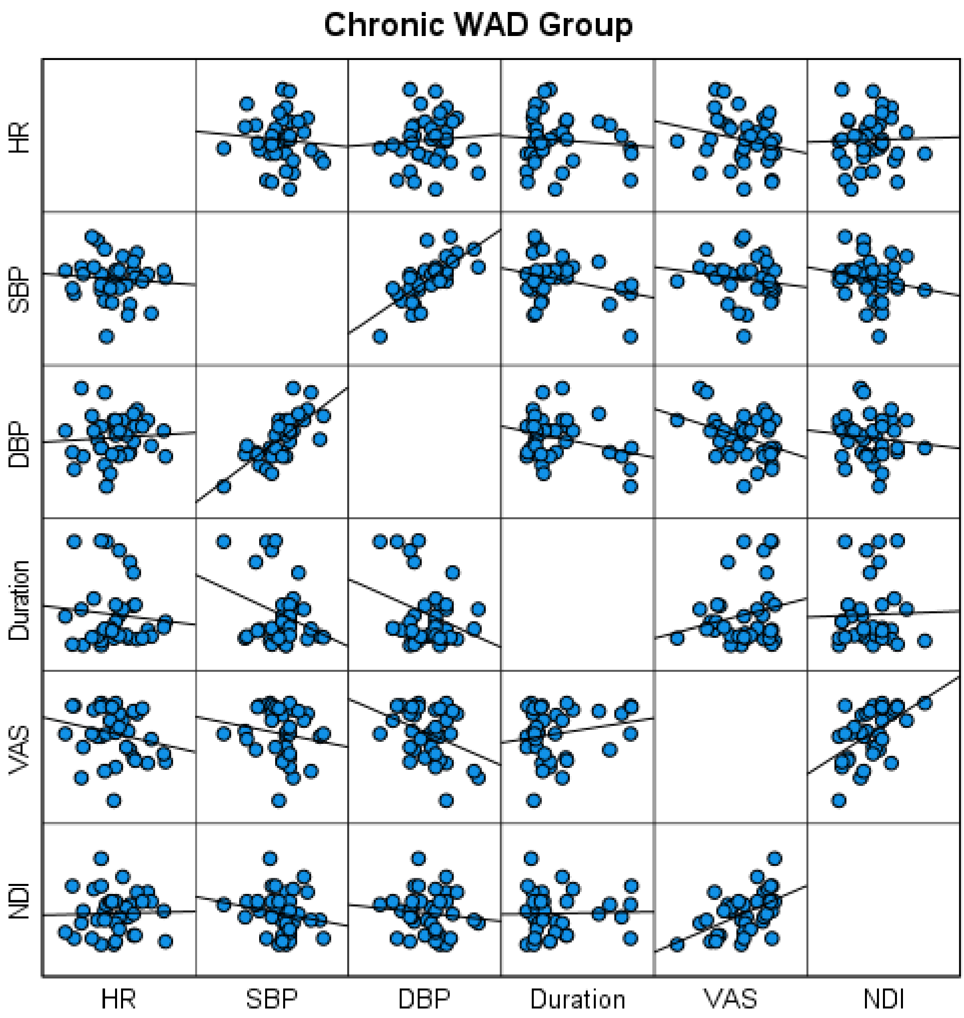

Supplementary Figure S1 Scatterplot matrix illustrating relationships between psychological variables, clinical characteristics and autonomic variables in people with chronic whiplash-associated disorder (WAD). HR = heart rate, SBP = systolic blood pressure, DBP = diastolic blood pressure, VAS = Visual Analogue Scale, NDI = Neck Disability Index, PCS = Pain Catastrophising Scale, TSK = Tampa Scale of Kinesiophobia, PCL-S = Posttraumatic Stress Disorder Checklist

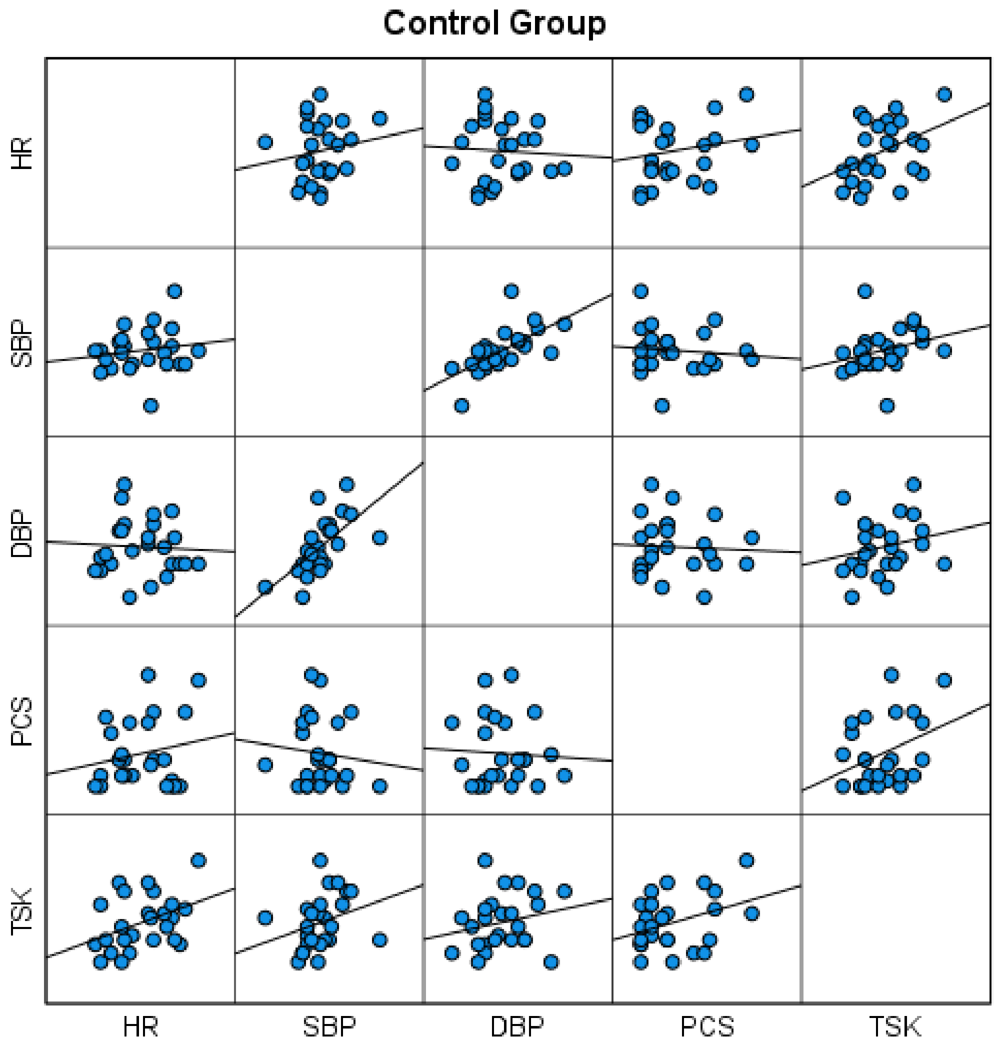

Supplementary Figure S2 Scatterplot matrix illustrating relationships between psychological and autonomic variables in pain-free control group. HR = heart rate, SBP = systolic blood pressure, DBP = diastolic blood pressure, PCS = Pain Catastrophising Scale, TSK = Tampa Scale of Kinesiophobia

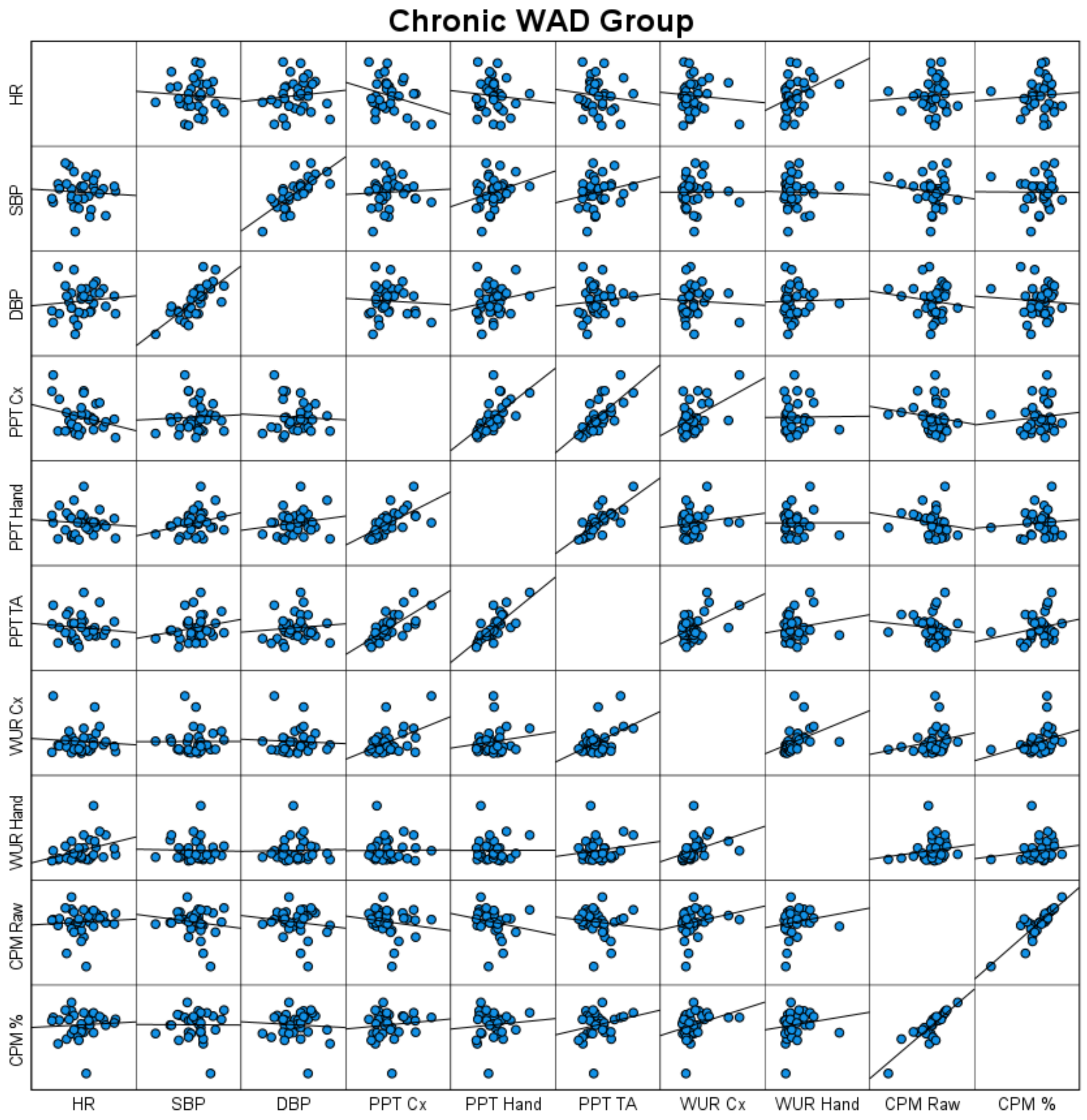

Supplementary Figure S3 Scatterplot matrix illustrating relationships between pain processing outcomes and autonomic variables in people with chronic whiplash-associated disorder (WAD). HR = heart rate, SBP = systolic blood pressure, DBP = diastolic blood pressure, PPT = pressure pain threshold, WUR = wind-up ratio, CPM = conditioned pain modulation (quantified in kPa and as % of baseline PPT), TA = tibialis anterior, Cx = cervical spine

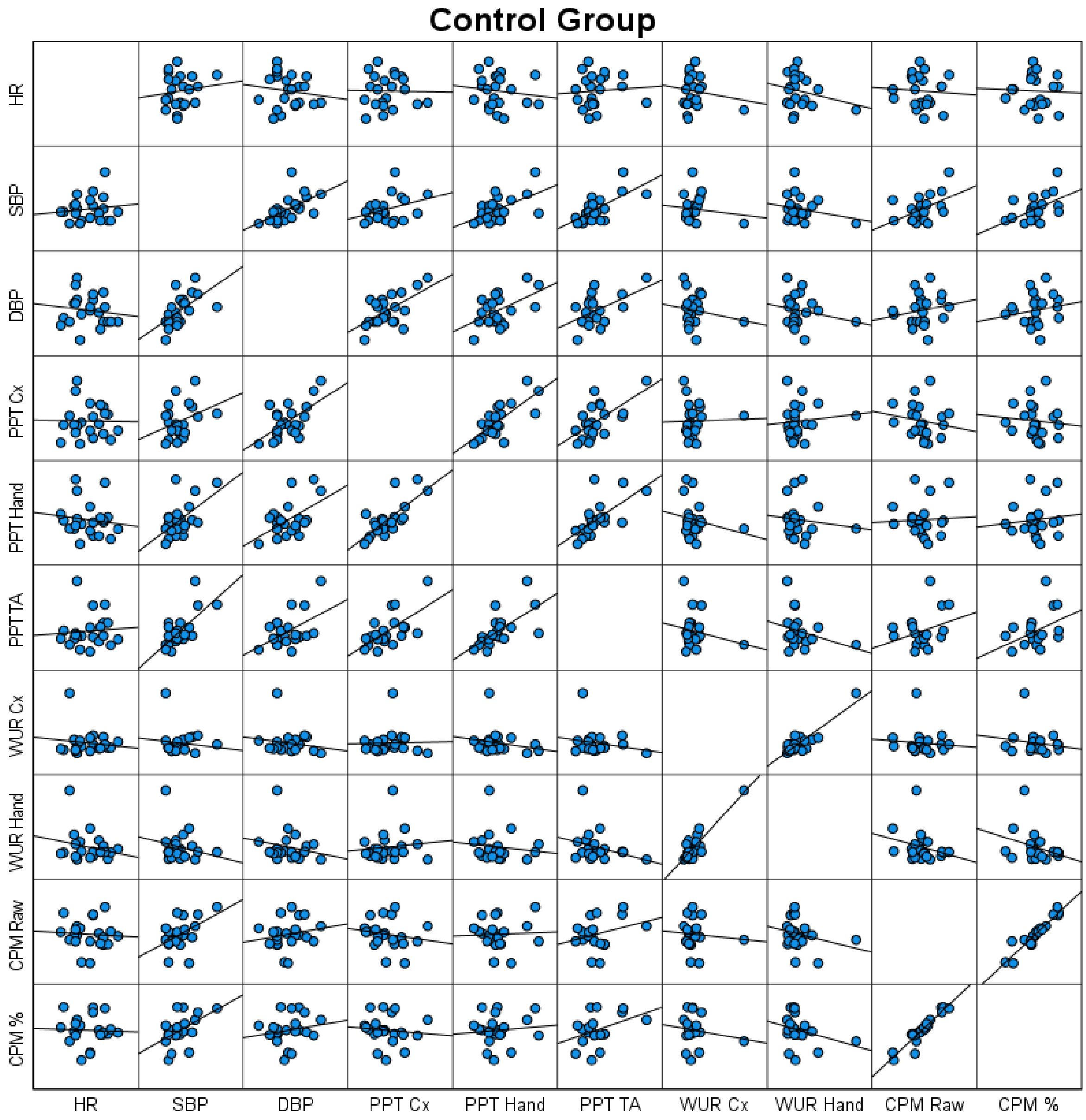

Supplementary Figure S4 Scatterplot matrix illustrating relationships between pain processing outcomes and autonomic variables in pain-free control group. HR = heart rate, SBP = systolic blood pressure, DBP = diastolic blood pressure, PPT = pressure pain threshold, WUR = wind-up ratio, CPM = conditioned pain modulation (quantified in kPa and as % of baseline PPT), TA = tibialis anterior, Cx = cervical spine
